# Non-invasive preoperative prediction of Edmondson-Steiner grade of hepatocellular carcinoma based on contrast-enhanced ultrasound using ensemble learning

**DOI:** 10.3389/fonc.2023.1116129

**Published:** 2023-07-05

**Authors:** Yao Wang, Dongbo Yuan, Hang Sun, Xiaoguang Pan, Fangnan Lu, Hong Li, Ying Huang, Shaoshan Tang

**Affiliations:** ^1^ Department of Ultrasound, Shengjing Hospital of China Medical University, Shenyang, Liaoning, China; ^2^ College of Medicine and Biological Information Engineering, Northeastern University, Shenyang, China; ^3^ School of Information Science and Engineering, Shenyang Ligong University, Shenyang, China; ^4^ Computer Science and Technology, School of Information and Control Engineering, Liaoning Petrochemical University, Fushun, China

**Keywords:** hepatocellular carcinoma, Edmondson-Steiner grade, contrast-enhanced ultrasound, radiomics, ensemble learning

## Abstract

**Purpose:**

This study aimed to explore the clinical value of non-invasive preoperative Edmondson-Steiner grade of hepatocellular carcinoma (HCC) using contrast-enhanced ultrasound (CEUS).

**Methods:**

212 cases of HCCs were retrospectively included, including 83 cases of high-grade HCCs and 129 cases of low-grade HCCs. Three representative CEUS images were selected from the arterial phase, portal vein phase, and delayed phase and stored in a 3-dimensional array. ITK-SNAP was used to segment the tumor lesions manually. The Radiomics method was conducted to extract high-dimensional features on these contrast-enhanced ultrasound images. Then the independent sample T-test and the Least Absolute Shrinkage and Selection Operator (LASSO) were employed to reduce the feature dimensions. The optimized features were modeled by a classifier based on ensemble learning, and the Edmondson Steiner grading was predicted in an independent testing set using this model.

**Results:**

A total of 1338 features were extracted from the 3D images. After the dimension reduction, 10 features were finally selected to establish the model. In the independent testing set, the integrated model performed best, with an AUC of 0.931.

**Conclusion:**

This study proposed an Edmondson-Steiner grading method for HCC with CEUS. The method has good classification performance on independent testing sets, which can provide quantitative analysis support for clinical decision-making.

## 1 Introduction

Hepatocellular carcinoma (HCC) is the sixth most common cancer in the world and the second most common cause of cancer-related death (1), and the incidence rate is increasing year by year (2). For early HCC patients, surgical resection is still an effective treatment (3). However, HCC is prone to relapse and metastasis, and the prognosis is poor (4, 5). Some studies have shown that recurrence is closely related to pathological manifestation (6). Several studies have shown that Edmondson-Steiner grade is an essential preoperative predictor of HCC (7, 8). For low-grade HCC with isolated lesions ≤ 2cm, the 3-year and 5-year relapse-free survival rates were 64% and 50%, respectively, while the 3-year and 5-year relapse-free survival rates for high-grade HCC were 39% and 29%, respectively (9, 10). According to the research, the recurrence rate of high-grade HCC is higher than that of low-grade HCC (11, 12). Moreover, compared with high-grade HCC, low-grade HCC has a higher surgical cure rate (13, 14) and higher short-term and long-term survival rates. Therefore, accurate prediction of the Edmondson-Steiner grade of HCC is highly significant for clinical decision-making, treatment plan optimization, and prognosis prediction (15–18).

Postoperative histopathological examination of resected tumor specimens is the gold standard for diagnosis and pathological grading of HCC (19). A needle biopsy can provide preliminary pathological grading before the operation. However, the risk of complications and misdiagnosis must always be considered (20). At present, some studies have shown that imaging examination may have the potential to reflect pathological grading (21, 22). MRI combined with liver-specific contrast agents, and diffusion-weighted imaging (DWI) has been proven to help evaluate the histological grading of HCC (23–25). Wu et al. showed that the radiomics characteristics of non-enhanced MRI and clinical factors helped predict the HCC grade before operation (26). The area under the ROC curve (AUC) of the best model for HCC grading prediction was 0.80. Jiseon et al. believed that CT texture analysis can provide texture features significantly related to higher tumor grade (27). Seitz et al. believe that CEUS and MRI have equal value for the differentiation and regulation of newly discovered liver tumors in clinical practice. Contrast-enhanced ultrasound (CEUS) and MRI are reliable for differentiating benign and malignant lesions and diagnosing hepatic hemangiomas and FNH. Characterization of metastasis and hepatocellular carcinoma is also very reliable (28). Compared with CT and MRI, CEUS provides dynamic perfusion images with fewer application restrictions (29). Liu et al. report that CEUS can give helpful information for the differential diagnosis of HCC, indicating its use in clinical practice (30). Wang et al. showed that the combination of the radiomics model of gray scale ultrasound and contrast-enhanced ultrasound with clinical information has potential clinical value for tumor grading (31). However, the quality of ultrasonic imaging depends significantly on the operator. Most of the previous studies are based on subjective feature recognition, which largely depends on the experience of doctors. These features may reveal pathological features that the naked eye can recognize (32).

Radiomics has been increasingly applied to the field of medical image analysis. It extracts massive amounts of information from radiological images for more in-depth mining, prediction, and analysis to help doctors diagnose more accurately. This study aimed to explore the value of sonographic features in predicting the Edmondson-Steiner grade of HCC before surgery.

## 2 Materials and methods

### 2.1 Materials

#### 2.1.1 Participants

The Shengjing Hospital of China Medical University Ethics Committee has approved this study. This study retrospectively collected the clinical information and ultrasonic images of 404 patients with primary HCC from April 2014 to March 2023. Among those, 212 cases were included in the first dataset, including 83 cases with high grades and 129 with low grades. An additional 51 cases, including 29 of low-grade and 22 of high-grade HCC, were used as an external independent testing set for evaluation. The inclusion criteria were as follows: (1) pathology confirmed HCC. (2) CEUS was performed one month before the operation, and the ultrasonic image data were complete. (3) No history of anti-tumor therapy such as liver transplantation (LT), microwave ablation (MWA), radiofrequency ablation (RFA), transcatheter arterial chemoembolization (TACE), etc. (4) The ultrasound image meets the analysis requirements (clear without artifacts and the target lesion is evident on the image). (5) No history of other malignant tumors. The exclusion criteria were: (1) liver cancer surgery and radiofrequency ablation before the operation; (2) Ultrasound images have severe artifacts, and tumors can hardly be observed with the naked eye.

We collected the preoperative laboratory examination information, including primary personal data (gender, age, hepatitis history), laboratory test results (alpha-fetoprotein (AFP), alanine aminotransferase (ALT), aspartate transaminase (AST), total bilirubin (TB), and prothrombin time (PT)).

**Figure 1:**
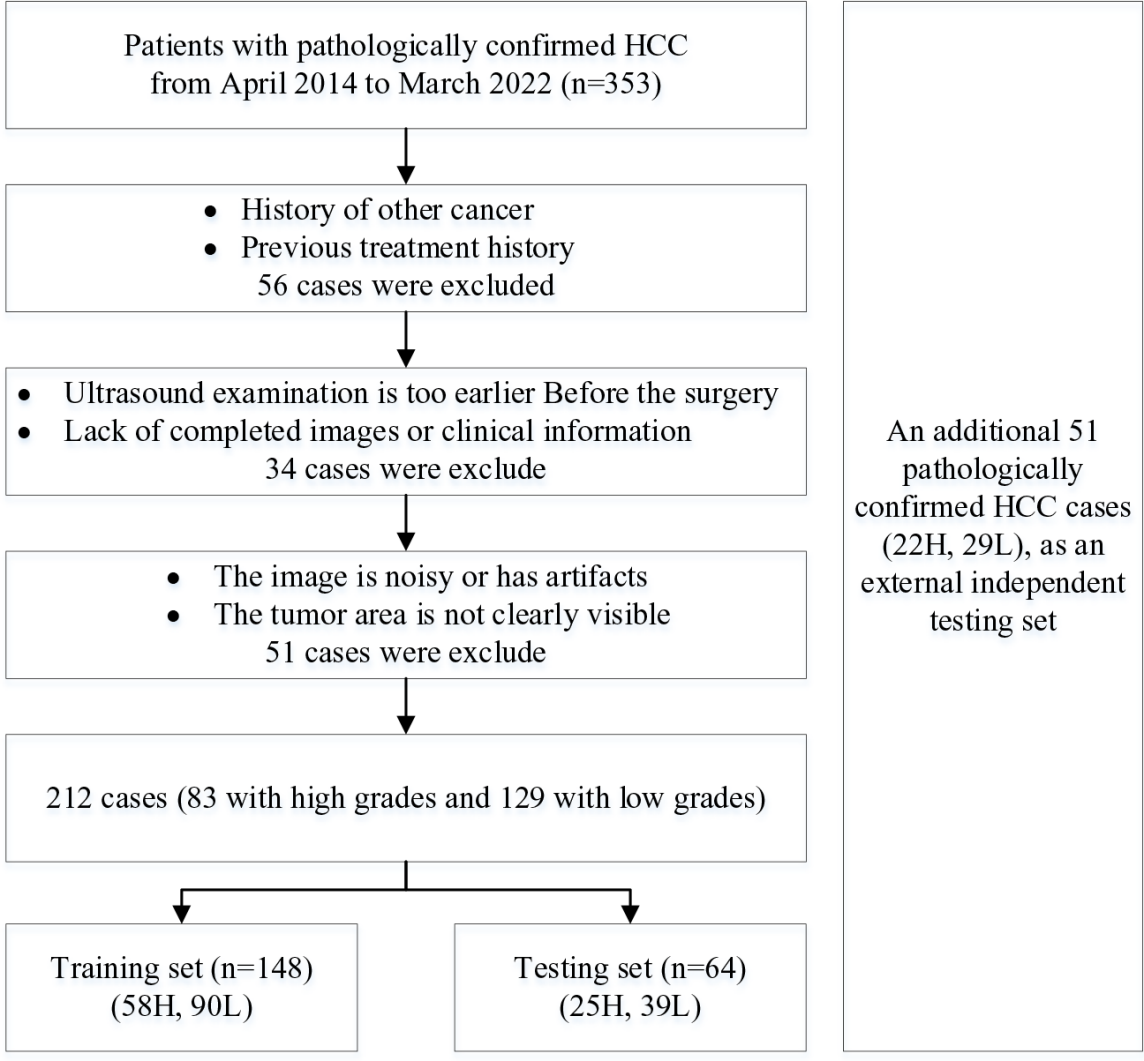
Inclusion and exclusion of patients. H stands for high-grade HCC, and L stand for low-grade HCC.

The differentiation grade of HCC reported by clinical pathology is based on the Edmondson-Steiner grade. In this study, low grades correspond to Edmondson grades I, I-II, and II, and high grades correspond to Edmondson grades III, III-IV, and IV. To summarize, 83 high-grade HCCs and 129 cases of low-level HCCs were included. Specific exclusion criteria is shown in **Figure 1**.

The patients were divided into training and testing cohorts randomly. There were 118 (79.7%) male patients and 30 (20.3%) female patients in the training cohort, while 51 (79.6%) male patients and 13 (20.4%) female patients were in the testing cohort. The median ages of patients in the training and testing cohorts were 58 years (range 29-79 years) and 57 years (range 28-74 years), respectively. In the training data set, the number of patients with low-grade HCC was 90 (60.8%), and 58 (39.2%) patients with high-grade HCC. In the testing data set, there were 39 (60.9%) cases with low-grade HCC and 25 (39.1%) cases with high-grade HCC. There was no significant difference between the training data set and the test data set in clinical pathological characteristics (p>0.05).

#### 2.1.2 CEUS acquisition

All CEUS examinations were performed by doctors with more than eight years of CEUS experience in liver cancer. All patients fasted for more than 8 hours before the examination. First, the whole liver was scanned with gray-scale ultrasound. The maximum diameter, echo signal, blood flow signal, shape, boundary, and lesion edge were evaluated and recorded. Store at least one original ultrasound image showing lesions and the same image containing measurement parameters in DICOM format. Then, the patient was given 2.4ml (3ml at most) of SonoVue (Bracco, Milan, Italy) *via* the anterior elbow vein, and then 5ml of 0.9% standard saline solution was added. The phase of contrast-enhanced ultrasound is divided into arterial phase, portal static phase, and delayed phase, which are 0-30s, 31-120s, and>120s after injection, respectively.

### 2.2 Methods

**Figure 2:**
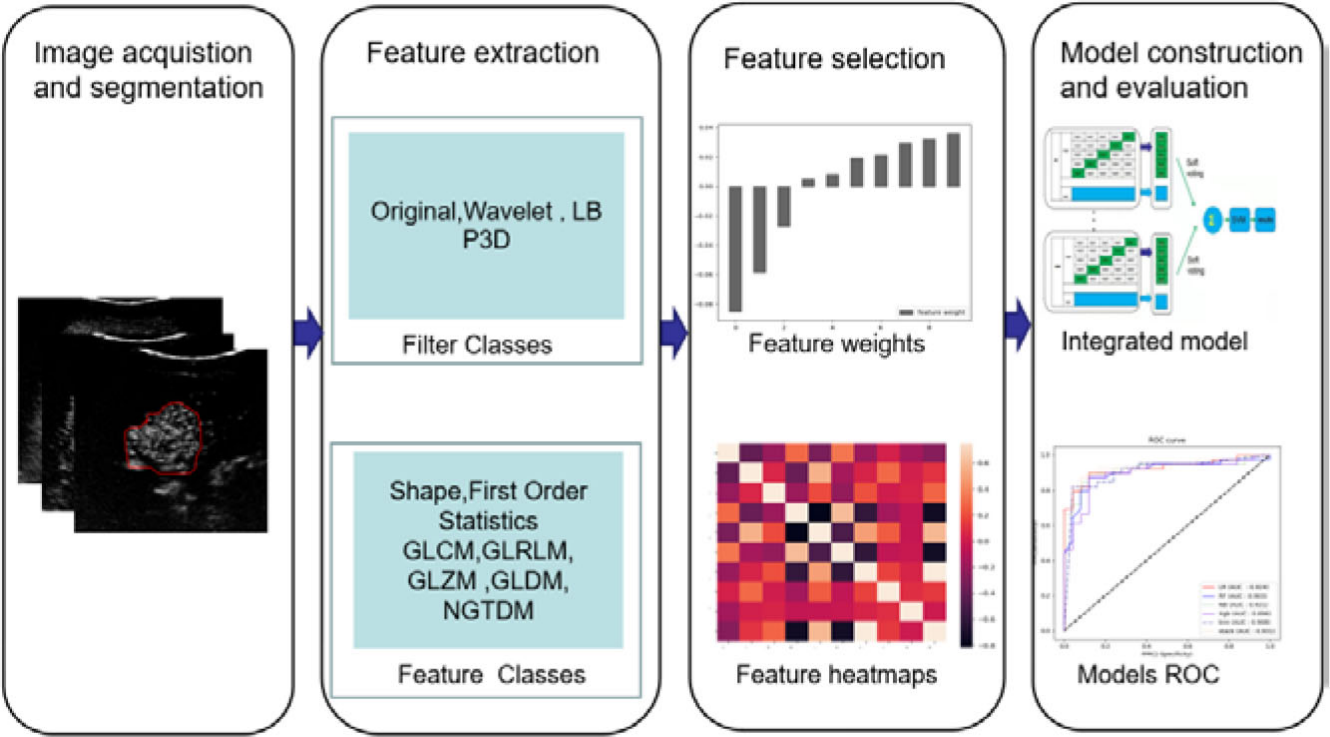
The research process includes image acquisition and segmentation, feature extraction and selection, model construction, and evaluation.

The static and real-time ultrasound images obtained in this study are retrieved from the picture archiving and communication system (PACS) and stored in DCM format. Three representative CEUS images were selected from the arterial phase (AP), portal vein phase (PVP), and delayed phase (DP) in which the image is clear shown and artifact-free. These images were stored in a 3-dimensional array. The main steps of this method can be described in **Figure 2**.

#### 2.2.1 Region of interest segmentation

We loaded the 3D image array into ITK-SNAP (v3.8.0, http://www.itk-snap.org) (33) for manual segmentation. To ensure the segmentation accuracy, a doctor with more than eight years of abdominal ultrasound experience and another doctor with ten years of abdominal ultrasound experience jointly complete the final confirmation.

#### 2.2.2 Feature extraction and selection

This study used the open-source software package Pyradiomic (https://www.radiomics.io/index.html) (34) for feature extraction and selection. We chose the original image, Local Binary Pattern (LBP) 3D, and the Wavelet for the filter classes. The feature classes extracted here include First Order Statistics, Shape-based (3D), Gray Level Co-occurrence (GLCM), Gray Level Run Length Matrix (GLRLM), Gray Level Size Zone Matrix (GLSZM), Gray Level Dependence Matrix (GLDM), Neighboring Gray Tone Difference Matrix (NGTDM). The independent sample t-test was used first for feature selection. Then, the Least Absolute Shrinkage and Selection Operator (LASSO) was used.

#### 2.2.3 Prediction model construction

212 patients were randomly divided into the training cohort (n=148) and the testing cohort (n=64). Based on the selected features, five single prediction models, namely linear regression (LR), random forest (RF), K-nearest neighbor (KNN), naive Bayes (NB), and Xgboost (XGB), were constructed by using a five-fold cross-validation method in the training set. Then, a support vector machine (SVM) was used as the meta-classifier to build the integrated prediction model.

#### 2.2.4 Model performance evaluation

This study used an independent testing set to evaluate the model’s performance. Sensitivity, specificity, accuracy, and AUC were used to evaluate the model’s performance.

## 3 Results

**Figure 3:**
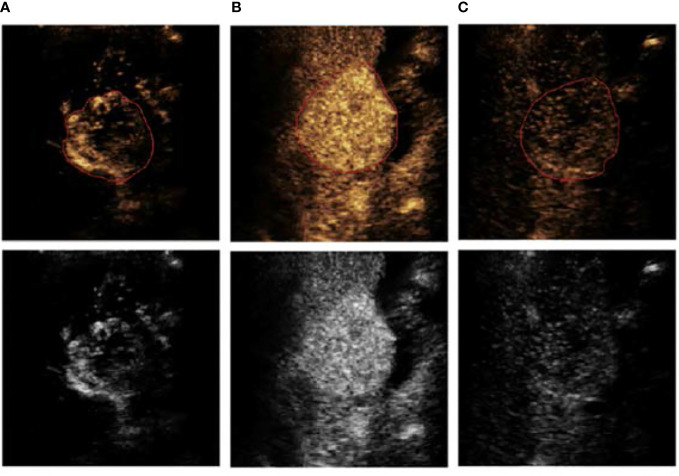
An example of ROI segmentation from a 63-year-old male patient with low-grade HCC, **(A)** arterial phase (AP), **(B)** portal vein phase (PVP), and **(C)** delayed phase (DP).

Experienced doctors conducted the ROI segmentation. **Figure 3** shows a segmentation example of a patient in different image phases.

**Figure 4:**
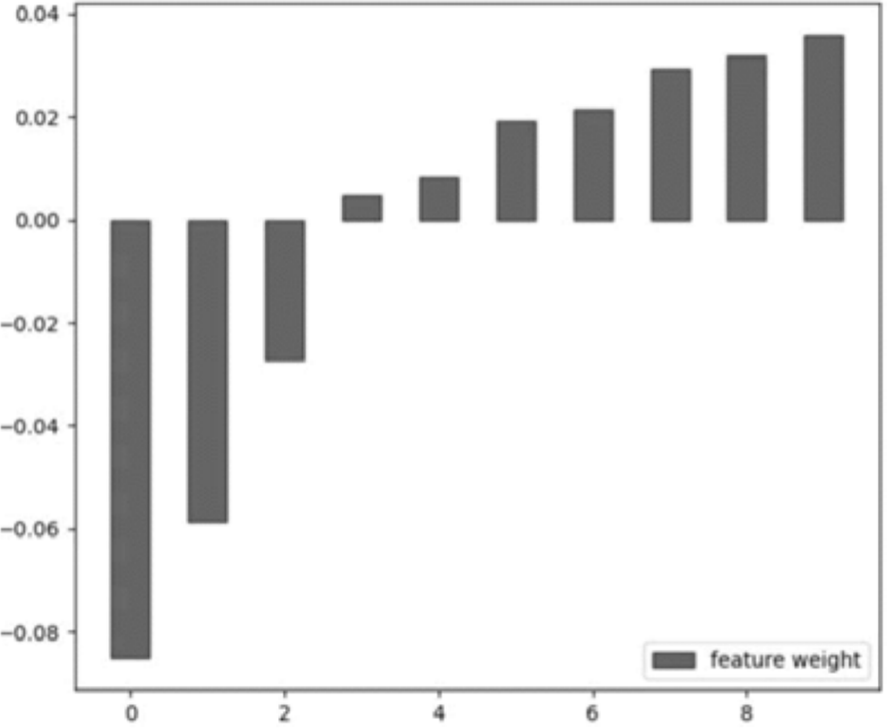
The selected feature weight. From left to right: original_glrlm_ShortRunLowGrayLevelEmphasis, LBP-3D-m1_firstorder_Kurtosis, LBP-3D-m2_glszm_GrayLevelNonUniformityNormalized, LBP-3D-k_glcm_Imc2, LBP-3D-k_glcm_Idn, log-sigma-1-0-mm-3D_glcm_Imc2, log-sigma-2-0-mm-3D_glcm_Idn, log-sigma-3-0-mm-3D_glszm_SmallAreaEmphasis, wavelet-LHH_glcm_Correlation, wavelet-LLL_glcm_Idn.

A total of 1338 features were extracted from the 3D CEUS images, and the number of selected features was 987 by the independent sample t-test. Then, after the selection by LASSO, ten features from seven categories were chosen finally: First Order Statistics, Shape-based (3D), GLCM, GLRLM, GLSZM, GLDM, and NGTDM. See **Figure 4**.

**Figure 5:**
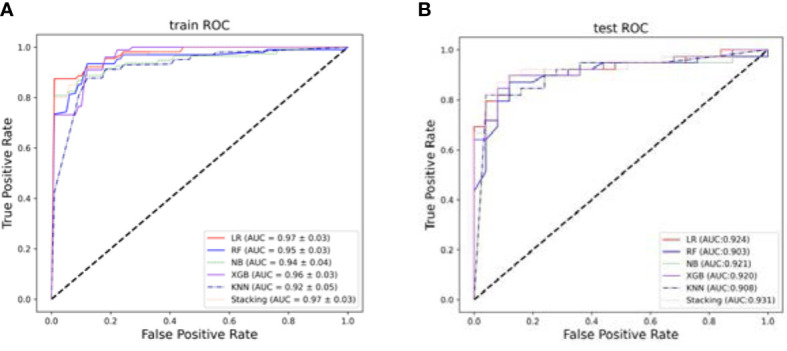
The ROCs. **(A)** The average performance of different models after five-fold cross-validation in the training set. **(B)** Models performance in the testing set.

Based on the selected features, five single prediction models, LR, RF, KNN, NB, and XGB, were constructed by using a five-fold cross-validation method in the training set. Then, SVM was used as the meta-classifier to build the integrated prediction model. **Table 1** shows the performance index in the training set, and **Table 2** shows the performance in the testing set of all the models. The corresponding ROCs can be found in **Figure 5**.

**Table 1:** Prediction performance of different models in the training set.

	Accuracy	Sensitivity	Specificity	AUC	F1-score
LR	0.899	0.933	0.901	0.970	0.914
RF	0.905	0.934	0.908	0.948	0.917
NB	0.844	0.847	0.881	0.941	0.861
XGB	0.905	0.941	0.909	0.960	0.920
KNN	0.885	0.917	0.887	0.921	0.898
Stacking	0.905	0.923	0.918	0.970	0.918

**Table 2:** Prediction performance of all the models in the testing set.

	Accuracy	Sensitivity	Specificity	AUC	F1-score
LR	0.875	0.897	0.897	0.924	0.897
RF	0.843	0.897	0.854	0.903	0.875
NB	0.875	0.897	0.897	0.921	0.897
XGB	0.859	0.872	0.895	0.896	0.883
KNN	0.843	0.897	0.854	0.908	0.875
**Stacking**	**0.891**	**0.897**	**0.921**	**0.931**	**0.909**

## 4 Discussion

Although significant progress has been made in treating HCC in recent years, the postoperative recurrence rate is still high, leading to poor clinical prognosis (35). Pathological grading is one of the critical factors in determining the postoperative recurrence of HCC. Compared with low-grade, high-grade HCC has a higher recurrence rate and shorter survival period, requiring expanded surgical margins and more frequent postoperative follow-up (36). Accurate preoperative prediction of pathological grading can provide a theoretical basis for individualized treatment decisions. However, the evaluation of HCC grading in clinical practice can only rely on pathological analysis of tumor tissue obtained after liver resection or puncture, which limits its value in guiding treatment decision-making before surgery.

Imaging examination is essential for diagnosing and preoperatively evaluating HCC. The commonly used contrast-enhanced imaging techniques in clinical practice include contrast-enhanced CT (CECT), contrast-enhanced MRI (CEMRI), hepatobiliary-specific MRI, and contrast-enhanced ultrasound (CEUS). Among the above four image types, CEUS differs from CECT, CEMRI, and hepatobiliary-specific MRI. The contrast agent of CEUS is a pure-blood pool contrast agent, which can accurately display the vascular architecture within the tumor. In addition, CEUS belongs to three-dimensional temporal imaging, which can reflect real-time changes in blood flow perfusion of tumor tissue (37). Previous studies have shown that factors such as tumor diameter, boundary, echo, clearance time, clearance degree, and LI-RADS classification of CEUS are related to the pathological grading of HCC, but their predictive ability is still weak (38–40).

Radiomics has been applied to the diagnosis of HCC and postoperative efficacy evaluation, and other aspects by analyzing existing images of patients without additional examinations or increasing the economic burden on patients (41, 42). This study used CEUS to predict the Edmondson-Steiner grading of HCC before operation. First, we extracted the Radiomics features from the enhanced three phases CEUS images and reduced the dimension of the features using independent sample t-test and LASSO. Next, five individual classifiers were trained using the five-fold cross-validation, and then the classifiers were integrated and obtained a prediction model with good performance.

The experimental results show that the prediction performance of the stacking ensemble model is much better than that of the individual classifier prediction model. For example, from **Table 2**, we can find that the accuracy and AUC of the ensemble model can reach 0.891 and 0.931, respectively.

**Figure 6:**
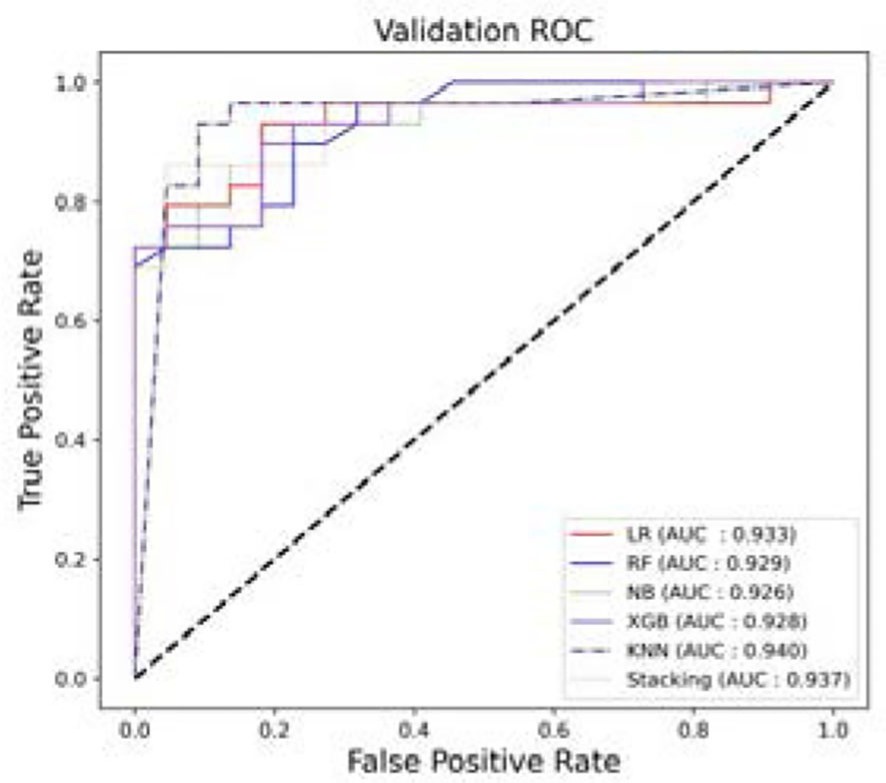
The ROCs. Models’ performance in the additional independent testing set.

The results of the additional testing set show that the performance of KNN compared to all other models, as shown in **Figure 6** and **Table 3**, has an accuracy and AUC of 0.901 and 0.940. The ensemble model also showed a good performance with an AUC of 0.937.

**Table 3:** Prediction performance of all the models in the additional independent testing set.

	Accuracy	Sensitivity	Specificity	AUC	F1-score
LR	0.843	0.931	0.818	0.932	0.807
RF	0.784	0.793	0.824	0.928	0.807
NB	0.823	0.862	0.833	0.926	0.847
XGB	0.843	0.965	0.838	0.927	0.866
KNN	0901	0.965	0.875	0.940	0.918
Stacking	0.823	0.896	0.812	0.937	0.852

Radiomics is a new field that aims to explore the potential relationship between medical images and tumor cell phenotypic characteristics in a non-invasive manner. According to previous studies, radiomics plays an essential role in tumor grading. **Table 4** lists recent research on the Edmondson-Steiner grading of HCC.

**Table 4:** Existing research results on the Edmondson-Steiner grading of HCC.

Method	Year	Median age (range)	Dataset cases	Feature selection	Model	EV	AC	AUC
Ref (10)	2021	24	193 (US)	Intra-class correlation coefficient , LASSO	SVM	Yes	0.818	0.849
Ref (16)	2020	57(28-74)	297(CECT)	T-test,ICC	XGBoost	No	0.7	0.801
Ref (18)	2019	55(25-74)	170(MRI)	LASSO	Logistic regression	No	0.761	0.8
Ref (31)	2021	TS:59(35.8), VS:24(35.3)	235(CEUS)	LASSO, ICC	SVM	No	0.757	0.785
Ref (43)	2022	TS:55(47-63),VS:56(47-62)	384(CECT)	Depth features	MSMR-DenseCNN	No	0.67	0.866
Ref (44)	2022	62(52-72)	137(MRI)	Random forest regression	Random forest	Yes	0.72	0.8
Proposed	2022	TS:58(29-79), VS:57(24-74)	212(CEUS)	T-test, LASSO	Stacking	Yes	0.823	0.937

TS stands for Training Set; VS stands for Validation set; EV stands for additional external testing set.

This study innovatively selected three representative phases of images from CEUS as the objects for feature extraction and analysis. By using ensemble learning methods, the classification performance has been significantly improved compared to existing studies. See **Table 4**.

Compared with enhanced CT and MRI, CEUS is real-time dynamic imaging, which has the advantages of low cost, safety, portability, and non-radiation, incomparable to other imaging technologies. The CEUS-based diagnosis is promising and can be used more widely in clinical practice.

However, our research has some limitations. First, we only classified HCC patients into two types: high-grade and low-grade. In the future study, the sample size will be further expanded, and the value of Radiomics for multi-category classification of HCC in CEUS will be further explored. Secondly, most of the enrolled patients had a history of viral hepatitis. The robustness of the proposed method needs to be verified in heterogeneous liver diseases with other high-risk factors of HCC. Third, we selected three phases of CEUS images by experienced doctors, which may have errors caused by subjective factors. Fourth, the ultrasound images came from a variety of different devices. The difference in ultrasonic parameters between patients could impact the experimental results. Fifth, our data comes from one center. In the future, we will take multi-center research to improve our experiments’ generalization performance.

## 5 Conclusion

This study proposed an automated Edmondson-Steiner grading method for HCC with CEUS. Based on three-dimensional dynamically enhanced ultrasound images, we used Radiomics methods to extract image bio-markers, made effective feature selection, and then used the integrated classifier to build a prediction model. The experimental results show that the method proposed in this study has good classification performance on independent testing sets, which can provide quantitative analysis support for clinical decision-making.

## Data availability statement

The raw data supporting the conclusions of this article will be made available by the authors, without undue reservation.

## Ethics statement

The studies involving human participants were reviewed and approved by the Shengjing Hospital of China Medical University Ethics Committee. The patients/participants provided their written informed consent to participate in this study.

## Author contributions

YW presented clinical problems, completed data collection, drawing segmentation labels, and establishing and evaluating models. DY and FL completed image data processing, model building and optimization. HS and XP helped applying the Radiomics and machine learning method. YH confirms the accuracy of segmentation labels and guides clinical related issues. HL and ST are responsible for the overall design and guidance. All authors contributed to the article and approved the submitted version.
